# MicroRNA-494 Regulates Endoplasmic Reticulum Stress in Endothelial Cells

**DOI:** 10.3389/fcell.2021.671461

**Published:** 2021-07-12

**Authors:** Namita Chatterjee, Eugenia Fraile-Bethencourt, Adrian Baris, Cristina Espinosa-Diez, Sudarshan Anand

**Affiliations:** ^1^Department of Cell, Developmental and Cancer Biology, Oregon Health and Science University, Portland, OR, United States; ^2^Department of Radiation Medicine, Knight Cancer Institute, Oregon Health and Science University, Portland, OR, United States

**Keywords:** microRNA, ER stress, endothelial cells, UPR – unfolded protein response, cell stress adaptation

## Abstract

Defects in stress responses are important contributors in many chronic conditions including cancer, cardiovascular disease, diabetes, and obesity-driven pathologies like non-alcoholic steatohepatitis (NASH). Specifically, endoplasmic reticulum (ER) stress is linked with these pathologies and control of ER stress can ameliorate tissue damage. MicroRNAs have a critical role in regulating diverse stress responses including ER stress. Here, we show that miR-494 plays a functional role during ER stress. Pharmacological ER stress inducers (tunicamycin (TCN) and thapsigargin) and hyperglycemia robustly increase the expression of miR-494 *in vitro.* ATF6 impacts the primary miR-494 levels whereas all three ER stress pathways are necessary for the increase in mature miR-494. Surprisingly, miR-494 pretreatment dampens the induction and magnitude of ER stress in response to TCN in endothelial cells and increases cell viability. Conversely, inhibition of miR-494 increases ER stress *de novo* and amplifies the effects of ER stress inducers. Using Mass Spectrometry (TMT-MS) we identified 23 proteins that are downregulated by both TCN and miR-494 in cultured human umbilical vein endothelial cells. Among these, we found 6 transcripts which harbor a putative miR-494 binding site. We validated the anti-apoptotic gene *BIRC5* (survivin) and *GINS4* as targets of miR-494 during ER stress. In summary, our data indicates that ER stress driven miR-494 may act in a feedback inhibitory loop to dampen downstream ER stress signaling.

## Introduction

The endoplasmic reticulum (ER) is the site of mRNA translation alongside proper folding and post-translational modifications of proteins destined for secretion or localization to various cellular membrane systems. In addition, the ER is the center of lipid biosynthesis, detoxification, homeostasis of intracellular Ca^2+^ and redox balance. Several pathologies including neurodegenerative diseases, diabetes, atherosclerosis and cancers have attributed ER stress as a critical driver of disease. When the folding capacity of the ER is challenged, the accumulation of un- or mis-folded proteins in the lumen of the ER triggers the unfolded protein response (UPR) (Ellgaard and Helenius, 2003; Ron and Walter, 2007). This response activates a trio of transducers: the PKR-like ER kinase (PERK), inositol requiring enzyme 1 α (IRE1α) and activating transcription factor 6 (ATF6), which work synergistically to control transcriptional and translational programs to either alleviate the burden of unfolded proteins and return to protein homeostasis or initiate apoptosis. It is thought that acute ER stress can trigger feedback mechanisms that protect cells from death by suppressing global translation and increasing ER chaperone levels, whereas persistent UPR activation or chronic unmitigated ER stress leads to increased oxidative stress, inflammation and eventual apoptosis (Zhang and Kaufman, 2008; Malhi and Kaufman, 2011).

Endothelial cells (ECs) encounter a variety of stressors and stimuli during development and disease (Brodsky and Goligorsky, 2012). Recent studies have implicated ER stress and the UPR as drivers of endothelial dysfunction in cardiovascular disease (Gargalovic et al., 2006; Lenna et al., 2014). For instance, ER stress is thought to promote both EC inflammation and apoptosis in atherosclerosis (Civelek et al., 2009; Zeng et al., 2009; Tabas, 2010). Furthermore, ER stress has been shown to contribute to vascular dysfunction and cardiac damage in preclinical models of hypertension (Kassan et al., 2012). Similarly, oxidative stress and ER stress pathways have been shown to be interlinked in ECs in metabolic syndromes such as diabetes and non-alcoholic fatty liver disease (NAFLD) (Zhou et al., 2012; Cao and Kaufman, 2014; Hammoutene and Rautou, 2019; Maamoun et al., 2019a,b). Therefore, ECs have adopted several mechanisms to regulate cell fate decisions in response to both acute and chronic ER stress (Cao and Kaufman, 2014).

MicroRNAs (miRs) are small non-coding RNAs that are critical regulators of physiological and pathological stress responses (Leung and Sharp, 2010; Olejniczak et al., 2018). We, and others have shown that specific miRs regulate EC responses to numerous effectors including: angiogenic growth factors (Wang et al., 2008; Shi et al., 2013), hypoxia (Ghosh et al., 2010; Voellenkle et al., 2012), DNA damage (Espinosa-Diez et al., 2018), and oxidative stress (Chen et al., 2015). Furthermore, specific miRs have been identified as modulators of ER stress in different models of disease (Upton et al., 2012; Maurel and Chevet, 2013; Sohn, 2018; Hiramatsu et al., 2020). Indeed Kassan et al. (2017) identified miR-204 as a promoter of ER stress in ECs by targeting the SIRT1 pathway. Our previous work identified a cohort of miRs that are induced by DNA damage in ECs (Wilson et al., 2016; Espinosa-Diez et al., 2018; Rana et al., 2020). We further characterized one of these miRs, miR-494, as a regulator of endothelial senescence in response to genotoxic stressors (Espinosa-Diez et al., 2018). In this study, we show that ER stress is a potent inducer of miR-494 likely via ATF6. Surprisingly, we find that miR-494 operates in a feedback loop to dampen the ER stress response potentially by targeting the anti-apoptotic protein survivin. Overall, our studies illuminate a novel function for miR-494 and open new avenues for further investigations into mechanisms by which miRs modulate stress responses.

## Materials and Methods

### Cell Culture and Reagents

Human umbilical vein endothelial cells (HUVECs) (Cat: NC9946677, Lonza) were cultured in EGM-2 media (Cat: CC-3162, Lonza) supplemented with all bullet kit components and 10% fetal bovine serum (Cat: FB-21, Lot# 649116, Hyclone) and were maintained at 37°C with 5% CO_2_. Cells used for experiments were low passage number (Gargalovic et al., 2006; Zhang and Kaufman, 2008; Tabas, 2010; Malhi and Kaufman, 2011; Brodsky and Goligorsky, 2012; Lenna et al., 2014). Tunicamycin (TCN) (Cat: T7765, Sigma) was dissolved in DMSO (Sigma). YM155 (Cat: S1130) was purchased from Selleckchem and dissolved in dH_2_O. Human Plasma Ox-LDL(Cat: L34357) was purchased from Thermo Fisher.

### Cell Transfection

HUVECs (50% confluence) were transfected with 50 nM mimic/inhibitor RNA using the lipofectamine RNAiMax reagent (Cat: 13778-150, Invitrogen) according to the manufacturer’s instructions. Specifically, mirVana miRNA Mimic Negative Control (Cat: 4464061, Ambion), hsa-miR-494-3p mirVana miRNA Mimic (MIMAT002816, Cat: 4404066, Assay ID MC12409, Ambion), mirVana miRNA Inhibitor Negative Control #1 (Cat: 4464076, Ambion), hsa-miR-494-3p mirVana miRNA Inhibitor (Cat: 4464084, Assay ID MH12409, Ambion), miRCURY LNA^TM^ Mimic Negative Control (Cat: YM00479903, Qiagen), and hsa-miR-494-3p miRCURY LNA^TM^ miRNA Mimic (MIMAT002816, Cat: YM00472106, Qiagen) were used for *in vitro* experiments.

For gene knock down, HUVECs were transfected with 10 nM siRNA using the lipofectamine RNAiMax reagent (Cat: 13778-150, Invitrogen) according to the manufacturer’s instructions. Specifically, siRNA for ATF6 (Cat: SR307883A, Origene), EIF2AK3 (Cat: SR306267A, Origene), ERN1 (Cat: SR301457A&B, Origene), BIRC5 (Cat: AM16708, Thermo Fisher), siRNA Negative Control (Cat: SR30001, Origene).

For plasmid transfections, HUVECs were transfected using the Cytofect HUVECs transfection kit (Cat:TF200K, Cell Applications). pEGFP-ATF6 was a gift from Ron Prywes (Addgene plasmid # 32955) (Chen et al., 2002).

### ER Stress and Cell Viability Assays

HUVECs were cultured as described in the cell culture section. Viability was assessed using a Cell-Titer Glo kit (Cat: G9241, Promega) per manufacturer’s instructions. For Hyperglycemia studies, cells were transfected and 6h later media was replaced with 1% FBS containing medium with no growth factors. Glucose (Cat: #A24940, Gibco) or Ox-LDL (Cat: L34357, ThermoFIsher) was added to this medium.

### Gene Expression

Total mRNA was isolated from cells and tissues using the miRNeasy Mini Kit (cat: 217004, Qiagen). Reverse transcription was performed using High Capacity cDNA Reverse Transcription Kit (Cat: 4368814, Applied Biosystems) according to the manufacturer’s instructions. Gene expression was measured using real-time quantitative PCR (qRT-PCR) with TaqMan^TM^ Master Mix II no UNG (Cat 4440049, Thermofisher Scientific) with the following primer/probe sets: miR-494-3p (Cat: 4427975, Assay ID: 002365), human primary miR-494 (Cat: 4427013, Assay ID: Hs04225959_pri) U6 snRNA (Cat: 4440887, Assay ID: 001973), sno234 (Cat: 4440887, Assay ID: 001234) DDIT3 (Cat: 4331182, Assay ID: Hs00358796_g1), BIRC5 (Cat: 4331182, Assay ID: Hs04194392_s1), XBP1 (Cat: 4331182, Assay ID: Hs00231936_m1) and GAPDH (Cat: 4351370, Assay ID: Hs02758991_g1) according to the manufacturer’s instructions. SYBR Green qRT-PCR assays were conducted using PowerUp SYBR Green Master Mix (Cat: A25741, Thermofisher Scientific) with primers shown in Table 1. Fold change was calculated using the 2^–ΔΔCt^ method relative to an internal control (GAPDH, β-Actin (ActB), sno234 or U6).

**TABLE 1:** Oligonucleotide primers used for SYBR green based qRT-PCR assays.

Primer	Sequence
mmu-DDIT3 For	CCA CCA CAC CTG AAA GCA GAA
mmu-DDIT3 Rev	AGG TGA AAG GCA GGG ACT CA
mmu-GAPDH For	GCC TGG TCA CCA GGG CTG C
mmu-GAPDH Rev	CTC GCT CCT GGA AGA TGG TGA TGG
hu-sXBP1 For	CTG AGT CCG AAT CAG GTG CAG
hs-sXBP1 Rev	ATC CAT GGG GAG ATG TTC TGG
hs-Total XBP1 For	TGG CCG GGT CTG CTG AGT CCG
hs-Total XBP1 Rev	ATC CAT GGG GAG ATG TTC TGG
hs-DDIT3 For	AGA ACC AGG AAA CGG AAA CAG A
hs-DDIT3 Rev	TCT CCT TCA TGC GCT GCT TT
hs-ERN1 For	TGC TTA AGG ACA TGG CTA CCA TCA
hs-ERN1 Rev	CTG GAA CTG CTG GTG CTG GA
hs-EIF2AK3 For	AAT GCC TGG GAC GTG GTG GC
hs-EIF2AK3 Rev	TGG TGG TGC TTC GAG CCA GG
hs-AFT6 For	ATG AAG TTG TGT CAG AGA ACC
hs-ATF6 Rev	CTC TTT AGC AGA AAA TCC TAG
hs-DUT For	TGC ACA GCT CAT TTG CGA ACG G
hs-DUT Rev	CCA GTG GAA CCA AAA CCT CCT G
hs-MINA For	ACT TTG GCT CCT TGG TTG G
hs-MINA Rev	CCC GGC TTC AGC ATA AAC
hs-DHFR For	CAT GGT TGG TTC GCT AAA CTG C
hs-DHFR Rev	GAG GTT GTG GTC ATT CTC TGG AAA TA
hs-GINS4 For	CCT AAC TCC TGC AGA GCT CAT T
hs-GINS4 Rev	AGG GGC AAA CTT TTC ATT CA
hs-UHRF1 For	CCA GCA GAG CAG CCT CAT C
hs-UHRF1 Rev	TCC TTG AGT GAC GCC AGG A
hs-BIRC5 For	GAC CAC CGC ATC TCT ACA TTC
hs-BIRC5 Rev	TGC TTT TTA TGT TCC TCT ATG GG
hs-ActB For	CCT GTA CGC CAA CAC AGT GC
hs-ActB Rev	ATA CTC CTG CTT GCT GAT CC
hs-GAPDH For	GAG TCA ACG GAT TTG GTC GT
hs-GAPDH Rev	TTG ATT TTG GAG GGA TCT CG

### Immunofluorescence

For immunofluorescence analysis, HUVECs were seeded (12,000 cells/well) on coverslips in a 24-well plate and transfected with mimic/inhibitor or appropriate control for 24h as described above. The following day, the media was aspirated and replaced with fresh media containing TCN (10 μg/mL TCN, Sigma) or DMSO (Sigma) for 24–48 h. The media was aspirated and cells were rinsed in ice cold PBS for 2 min followed by fixation in 4% paraformaldehyde diluted in PBS (Cat: NC9658705, FisherSci) for 10 min at room temperature (RT). Coverslips were rinsed in PBS three times and incubated in serum free DAKO Protein Block (Cat: X0909, DAKO) for 1h at RT. Next, coverslips were incubated overnight at 4°C in anti-survivin primary antibody diluted in PBS/5% BSA. After rinsing in PBS three times, the coverslips were incubated in the dark with Alexa Fluor^®^ 488 goat anti-rabbit secondary antibody diluted in PBS/5% BSA. Refer to Table 2 for antibody information and dilutions. After rinsing in PBS three times, the coverslips were mounted onto glass slides with Aqua-Poly/Mount (Cat: 18606, Polysciences Inc.). Slides were left to cure overnight at RT prior to imaging. Coverslips were imaged using a Nikon/Yokogawa CSU-W1 Spinning Disk Confocal Microscope. All imaging settings remained constant throughout the imaging sessions. The percentage stained area was analyzed using the open-access Image J software (NIH).

**TABLE 2:** Antibodies used in this study.

Antibody	Source	Identifier	Dilution
Survivin	Cell signaling technology	2,808	1:25
CHOP	Novus	NBP2-66856	1:250
XBP1-s	Cell signaling technology	83,418	1:25
GINS4	Novus	NBP2-16659	1:25
GAPDH	Cell signaling technology	21,18S	1:1000
IRDye^®^ 800CW Goat anti-Rabbit IgG (H + L)	Li-Cor	926–32,211	1:5000
Alexa Fluor^®^ 488 (goat anti-rabbit)	Cell signaling technology	4,412	1:100

### Western and Simple Western Blots

HUVECs were seeded in six well plates (2,00,000 cells/well) and transfected as described above. After 24 h, the media was removed and cells were treated with 10 μg/mL TCN for 48 h. After treatment, the media was aspirated and the cells were washed twice in ice cold PBS and lysed directly in the plate in RIPA buffer (Cat: PI89900, Pierce) containing Protease Inhibitor Mini Tablets (1/10 mL RIPA buffer, Cat: A322953, Pierce) with phosphatase inhibitor cocktail 2 and 3 (1:1000, Cat: P5726 and P0044) for 15 min on ice. Lysates were rotated at 4°C for 30 min-2 h and then centrifuged at 12,000 x *g* at 4°C for 40 min. The supernatant was collected and protein concentration was determined using the Pierce BCA Protein assay kit (Cat: 23225). Lysates from the liver were obtained by tissue homogenization following the addition of RIPA buffer and centrifugation as described before. For western blot, samples were mixed with 4X Protein Sample Loading Buffer (Li-Cor; Cat: 928-40004) supplemented with 5% of beta-mercaptoethanol, denaturalized and loaded in 4–20% precast polyacrylamide gels (BioRad: cat: 4561094). Electrophoresis was done in 1× Tris/Glycine/SDS at 200 V during 30–40 min. Trans-blot Turbo Transfer system (BioRad) was used to transfer the proteins to a PVDF membrane following the manufacture instructions. Blocking and antibody dilutions were done in Intercept^®^ (TBS) Blocking Buffer (BioRad; Cat: P/N 927-60001). The membranes were developed using Li-Cor Odyssey Clx imaging system. For simple western blot, samples were diluted to 0.75 μg/mL with 1× Sample Buffer (ProteinSimple). Protein quantification was performed using a 12–230 kDa 25 lane plate (Cat: PS-MK15; ProteinSimple) in a ProteinSimple Wes Capillary Western Blot analyzer according to the manufacturer’s instructions. The standard Simple Western protocol was altered to increase sample load time to 13s and a separation time of 33 min.

### TMT-Mass Spectrometry

Tandem mass tag (TMT) labeling and mass spectrometry were performed by the OHSU proteomics core facility as previously described (Plubell et al., 2017). Briefly, HUVECs were treated with miRs (*n* = 3 biological replicates) or TCN (*n* = 2, biological replicates) as described above. After 24–48 h, samples were lysed in 50 mM triethyl ammonium bicarbonate (TEAB) buffer (50 μg of protein/sample) followed by a Protease Max digestion, a microspin solid phase extraction and TMT 10-plex labeling according to manufacturer’s protocol (Thermo Scientific). Multiplexed TMT-labeled samples were separated by two-dimensional reverse-phase liquid chromatography (18 fractions). Tandem mass spectrometry data was collected using an Orbitrap Fusion Tribrid instrument (Thermo Scientific) using the default SPS MS3 method, also described in Ref. 32. RAW instrument files were processed using Proteome Discoverer (PD) (version 1.4, Thermo Scientific). SEQUEST searches used a Swiss-Prot human protein database (20,120 sequences, release 2016.10) with reversed-sequence decoy strategy to control peptide false discovery, and identifications were validated by Percolator software (q-score less than 0.05). Key search parameters were trypsin cleavage with up to two missed cleavages, monoisotopic parent ion mass tolerance of 1.25 Da, monoisotopic fragment ion tolerance of 1.0005 Da, variable oxidized methionine modifications, and fixed alkylated cysteine and TMT regent labels (on N-terminus and lysines). Search results were exported from PD and TMT reporter ion intensities were processed with in-house scripts available at https://github.com/pwilmart/PAW_pipeline. Differential protein abundance was determined using the Bioconductor package edgeR and detailed in a results spreadsheet in the Supplementary Material.

### Statistical Analysis

All statistical analysis was performed using Prism software (GraphPad Software, San Diego, CA, United States). Differences between pairs of groups were analyzed by Student’s *t*-test. Comparison among multiple groups was performed by one-way ANOVA followed by a *post hoc* test (Tukey’s or Holm-Sidak). In the absence of multiple comparisons, Fisher’s LSD test was used. Values of *n* refer to the number of experiments used to obtain each value. For mouse studies where the data was not normally distributed, we used two-tailed Mann–Whitney *U* test. Values of *p* ≤ 0.05 were considered significant.

## Results

### ER Stress Induces miR-494 *in vitro*

**FIGURE 1:**
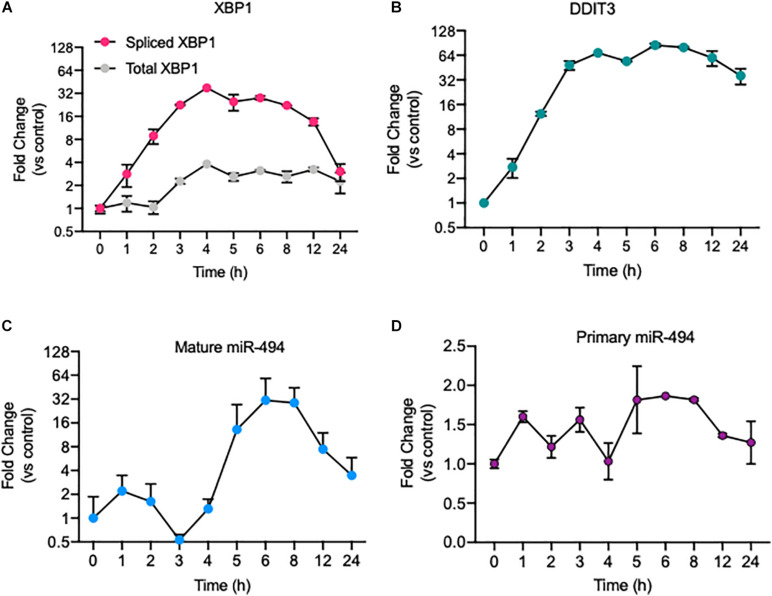
ER stress induces expression of the primary and mature forms of miR-494. Relative mRNA expression of ER stress responsive genes as measured using qRT-PCR. **(A)** spliced *XBP1* and Total *XBP1* and **(B)** *DDIT3* (CHOP) mRNA levels in HUVECs treated with 5 μg/mL tunicamycin (TCN) over a time course. Relative expression of **(C)** mature miR-494 and **(D)** primary miR-494 (pri-miR-494) in HUVECs treated with TCN over a time course. Gene expression is normalized to GAPDH or U6 and mean fold change compared to vehicle control or time 0h is shown. Graphs are representative of one biological replicate from three independent replicates where values indicate mean ± standard deviation.

We previously showed that miR-494 is responsive to radiation and chemical inducers of genotoxic stress and functions to increase endothelial senescence during DNA damage responses (Espinosa-Diez et al., 2018). Given the intricate relationship between radiation, oxidative stress and ER stress (Cao and Kaufman, 2014; Maamoun et al., 2019b), we asked if ER stress affected miR-494 expression and function. First, we confirmed a robust ER stress response to known inducers, TCN and thapsigargin (TG), in HUVECs by measuring the level of the transcription factors spliced *XBP1* (mRNA: *sXBP1*, protein: XBP1s) and *DDIT3* (CHOP), which are well characterized markers of the ER stress response (Figures 1A,B). We observed that TCN significantly increased the levels of mature miR-494 (Figure 1C) and to a lesser extent, the primary unprocessed miR-494 transcript (Figure 1D). Similarly, TG also induced *sXBP1* and *DDIT3* in parallel with the primary and mature forms of miR-494 (Supplementary Figure 1). We also saw a comparable increase in both DDIT3 and the primary miR-494 transcript in another EC line (Human Microvascular Endothelial cells - HMVECs) (Supplementary Figure 2). Physiological inducers of ER stress, hyperglycemia and to a lesser extend Ox-LDL treatment also induced miR-494 expression changes in these cells in the absence of growth factors and in low serum culture conditions (Supplementary Figure 3).

A triumvirate of transducers (ATF6, IRE1α & PERK) co-ordinate ER stress response programs to promote cell recovery, or in the case of excessive or chronic stress, initiate apoptosis. We asked which of these transducers was responsible for miR-494 induction in response to ER stress using siRNAs (Supplementary Figure 4A). We found that knockdown of ATF6 but not IRE1α (*ERN1*) or PERK (*EIF2AK3*) significantly decreased the induction of primary miR-494 6h after TCN treatment (Supplementary Figure 4B). In contrast, the mature miR-494 increase disappeared when any of these pathways was knocked down (Supplementary Figure 4C). Ectopic expression of ATF6 in HUVECs modestly increased the levels of primary miR-494 (Supplementary Figure 4D) although our transfection efficiency was <50%. Therefore, these data indicate that while the steady state levels of primary miR-494 are dependent on ATF6 but the mature miR-494 levels are dependent on all three ER transducers.

### miR-494 Diminishes ER Stress

**FIGURE 2:**
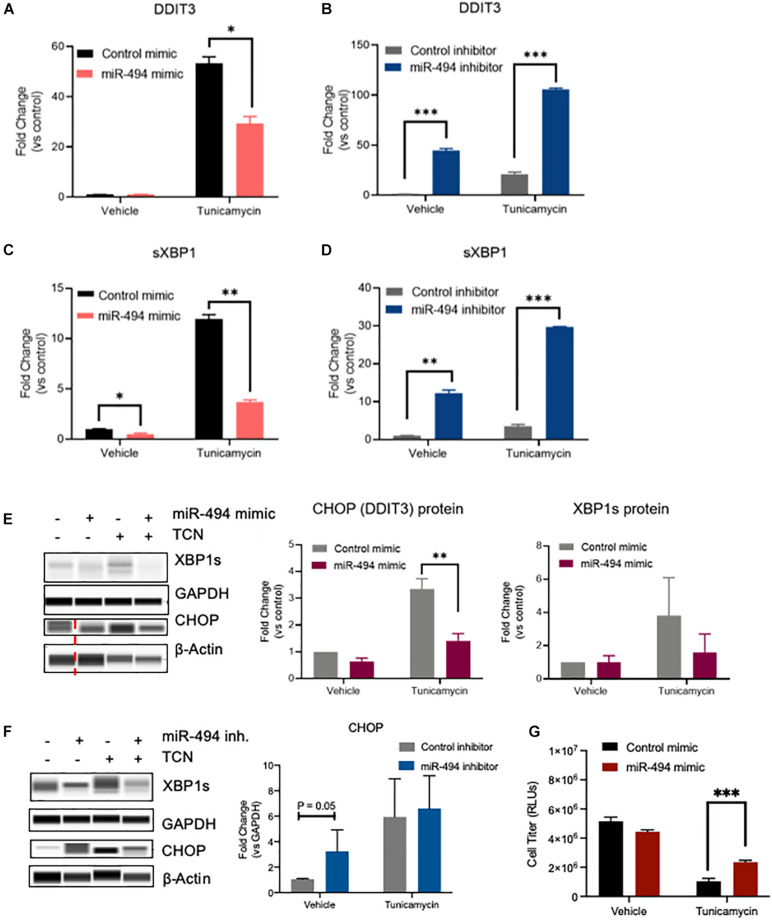
miR-494 is a negative regulator of ER stress *in vitro*. Relative mRNA expression of ER stress responsive genes as measured by qRT-PCR. **(A,B)** *DDIT3* (CHOP), **(C,D)** spliced *XBP1* in HUVECs treated with 10 μg/mL TCN 48h after transfection with **(A,C)** miR-494 mimic or **(B,D)** miR-494 inhibitor. Gene expression is normalized to GAPDH and mean fold changes compared to control treatments are shown. **(E,F)** Simple Western blot analysis of HUVECs transfected with miR-494 mimic or control (24 h) **(E)** or miR-494 inhibitor or control **(F)** followed by TCN (10 μg/mL) for 24 h. **(G)** Cell viability in HUVECs as treated in **(A)** Vertical dotted red line indicates non-adjacent lanes. Graphs are mean + SEM fold changes of biological replicates from *n* = 3 independent experiments. ^∗^*P* < 0.05, ^∗∗^*P* < 0.01, ^∗∗∗^*P* < 0.001 by two-tailed Student’s *T*-test.

To understand the functional relevance of miR-494 during ER stress, we performed gain- and loss-of function experiments utilizing miR-494 mimics or inhibitors, respectively. We confirmed that a miR-494 mimic significantly elevated miR-494 levels in HUVECs and conversely, miR-494 inhibitor decreased endogenous miR-494 levels (Supplementary Figure 5). Pretreatment of HUVECs with miR-494 mimic suppressed the TCN based induction of the ER stress responsive gene *DDIT3* (Figure 2A). Conversely, inhibition of miR-494 robustly increased levels of *DDIT3* (Figure 2B). Similarly, miR-494 mimic decreased levels of *sXBP1* (Figure 2C) whereas, inhibition of miR-494 increased *sXBP1* mRNA both *de novo* and in combination with TCN (Figure 2D). We validated that the miR-494 mediated changes in these mRNAs also persisted at the protein level (Figures 2E,F) with the caveat that the impact of the miR is more substantial on the mRNA levels compared to the protein levels. Consistent with the decrease in ER stress, the miR-494 mimic conferred modest but measurable protection to HUVECs from TCN induced cell death (Figure 2G). Taken together, our data suggest that miR-494 diminishes the induction of ER stress-associated transcription factors and plays a protective role in HUVECs during ER stress.

### Mass Spectrometry Identifies Putative Targets of miR-494 Relevant for ER Stress

**FIGURE 3:**
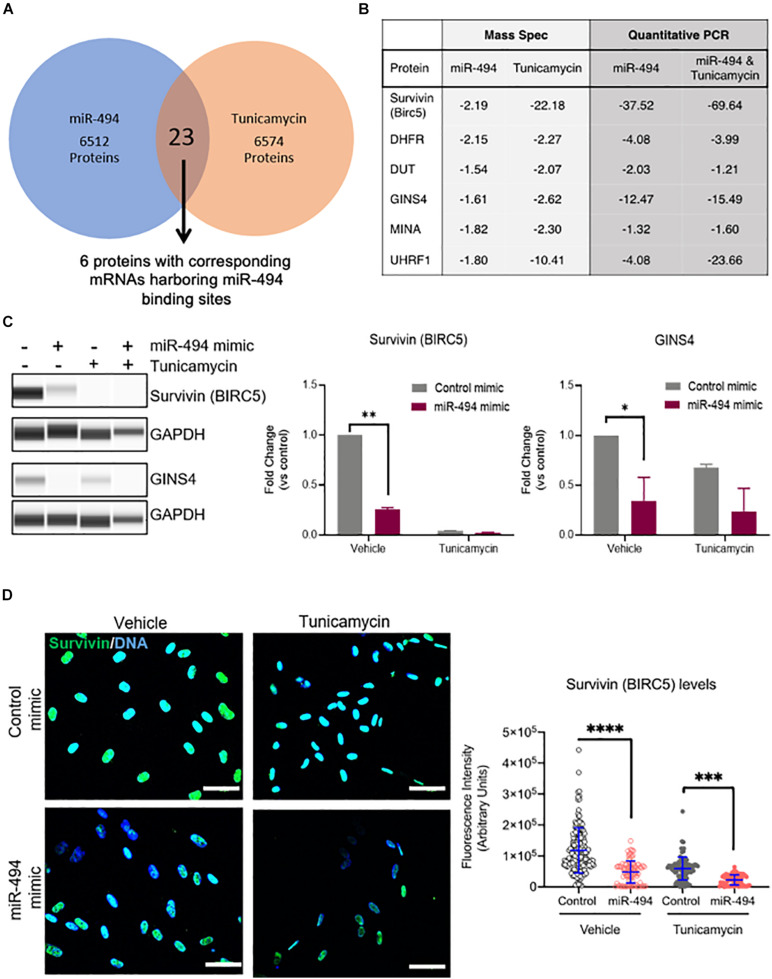
miR-494 regulates target genes in cell survival and DNA replication. **(A)** Venn diagram showing the number of downregulated target proteins in a Tandem Mass Tag labeled Mass Spectrometry profile from HUVECs treated with TCN or transfected with miR-494 compared to vehicle treatment or control miR respectively. **(B)** Fold-change (compared to respective controls) of protein or mRNA levels as assessed by Mass Spectrometry or qRT-PCR respectively for the six targets that were downregulated in both TCN and miR-494 groups. All six targets harbor miR-494 binding sites in their 3′UTRs. **(C)** Representative Simple Western blot showing survivin (*BIRC5*) and GINS4 levels in HUVECs 24 h after miR-494 transfection followed by TCN treatment (24 h). Right panels show quantitation of biological replicates. ^∗^*P* < 0.05, ^∗∗^*P* < 0.01, by two-tailed Student’s *T*-test. **(D)** Immunofluorescence images showing survivin expression in HUVECs 24 h after miR transfection and/or TCN treatment. Right panel shows quantification via ImageJ of survivin fluorescence intensity in each cell. Each dot represents individual cells. Scale bar in white = 50 μm. ^∗^*P* < 0.05, ^∗∗^*P* < 0.01, ^∗∗∗^*P* < 0.001 by one-way ANOVA with *post hoc* Tukey’s correction.

miRs typically are thought to regulate numerous targets in a context dependent manner. We have previously shown that miR-494 targets the Mre11a, Rad50, Nbn (MRN) complex in the DNA damage repair pathway in response to genotoxic stressors (Espinosa-Diez et al., 2018). To identify the targets relevant for miR-494 in the context of ER stress, we undertook a proteomics-based approach. We used Tandem mass tag-based mass spectrometry (TMT-MS) to compare changes in the proteome with either miR-494 mimic treatment or TCN treatment. A complete dataset is available from the PRIDE database (Vizcaino et al., 2013) accession number PXD019992. We found 23 proteins were commonly downregulated between the TCN and miR-494 treatment conditions (Figure 3A and Supplementary Figure 6). Among these, we identified six proteins whose mRNAs harbored putative miR-494 binding sites in their 3′UTRs as predicted by the TargetScan algorithm (Agarwal et al., 2015). We validated the expression of these six genes using qRT-PCR (Figure 3B and Supplementary Figures 7A–D). All six targets were validated and demonstrated a substantial decrease in expression in response to miR-494 gain-of-function activity. Conversely, inhibition of miR-494 restored *BIRC5*, *GINS4, MINA* and to a lesser extent, *UHRF1* mRNA levels in HUVECs treated with TCN (Supplementary Figure 7E). Similarly, inhibition of miR-494 also increased GINS4 protein levels (Supplementary Figure 7F) whereas the TCN treatment of miR-494 inhibited cells led to significantly more cell death precluding accurate estimation of protein levels at 48h post treatment. Since *BIRC5* and *GINS4* were consistently regulated in a miR-494 dependent fashion, we chose to further validate these two targets at the level of protein expression. We found survivin (gene: *BIRC5*), and GINS4 protein levels were significantly decreased upon treatment with miR-494 mimic alone, TCN treatment alone, and the sequential combination of miR pre-treatment (24 h) followed by TCN (24 h) (Figure 3C). Finally, we used immunofluorescence staining and confocal microscopy to evaluate survivin expression in HUVECs. Consistent with our western blot data, we observed a significant decrease in the amount of survivin in cells transfected with either miR-494 mimic or TCN treatment alone or in the sequential combination (Figure 3D). Multiple studies have demonstrated that miR-494 binds to the 3′UTR and leads to subsequent degradation of the survivin (*BIRC5*) transcript (Zhu et al., 2015; Xu et al., 2018; Yun et al., 2018). However, to our surprise, genetic or pharmacological disruption of survivin did not significantly impact the induction of DDIT3 mRNA in response to TCN (Supplementary Figure 8). This might suggest perhaps the decrease of survivin alone may not be sufficient to protect against TCN induced DDIT3 transcription. Alternatively, it is also possible that the miR-494 induced decrease in DDIT3 is independent of survivin and might function through other target pathways.

## Discussion

**FIGURE 4:**
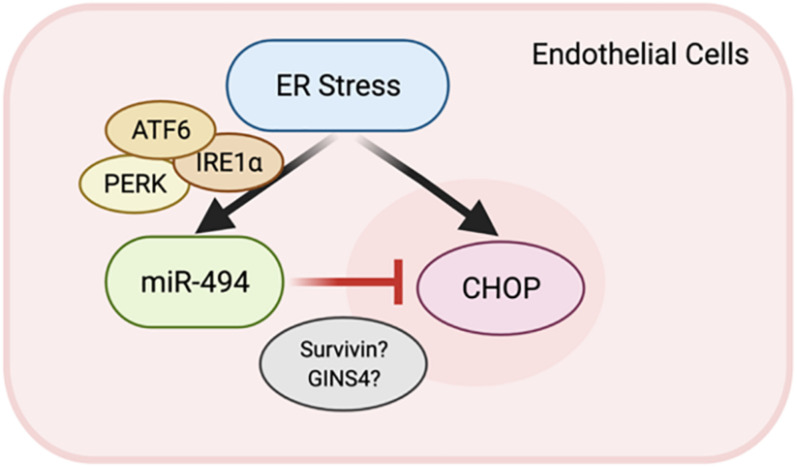
A feedback loop between miR-494 and ER stress. Schematic showing the relationship where ER stress induction of miR-494 inhibits *DDIT3* and functions to dampen ER stress via modulation of target genes including *BIRC5* and *GINS4*.

Here, we show that ER stress is a potent inducer of miR-494 in endothelial cells (ECs) *in vitro*. Our data indicates that the ER stress-associated increase in expression of miR-494 occurs in an ATF6 dependent manner. Upon induction, miR-494 decreases the level of a group of six genes important in DNA replication, cell proliferation and viability. We show that miR-494 gain-of-function diminishes the magnitude of the UPR induced by TCN. Overall, this report shows that ER stress induces both DDIT3 and miR-494 which downregulates DDIT3 to dampen the ER stress response by mechanisms which have yet to be revealed (Figure 4).

Many chronic human diseases have an established link within the ER stress response signaling cascades. Mechanistically, ER stress and the UPR are triggered by the accumulation of misfolded proteins (Ron and Walter, 2007; Wang and Kaufman, 2016). The UPR is initiated by the dissociation of the molecular chaperone protein BiP from the three sensors, PERK, IRE1α and ATF6. Dissociation of BiP activates these proteins resulting either oligomerization or export from the ER. These activated sensors transduce signals to initiate pathways which work in a coordinated manner to halt protein translation, increase ER chaperone levels and clear misfolded proteins. Known chemical inducers of ER stress, TCN and thapsigargin (TG) generate a robust UPR in multiple models of disease including atherosclerosis and NASH (Witte and Horke, 2011; Lee et al., 2012). TCN is a toxic aminoglycoside antibiotic which triggers ER stress by preventing core oligosaccharide addition to nascent polypeptides, thereby blocking proper protein folding. Numerous studies have shown that TCN stimulates all three of the UPR signaling pathways causing an upsurge in transcription factor activity (e.g., CHOP, XBP1 splicing), global inhibition of translation (eIF2α phosphorylation), and ATF6 translocation to the Golgi apparatus. We found that TCN treatment of ECs induced CHOP transcription and XBP1 splicing, which are indicative of an appropriate ER stress response. Further, TCN consistently generated a rapid and robust upregulation of miR-494 in multiple EC cell lines (Figure 1 and Supplementary Figure 2). TG, a non-competitive inhibitor of the Sarco/Endoplasmic Reticulum Calcium ATPase (SERCA) disrupts calcium homeostasis in the ER. In our experiments, TG treated ECs experienced similar signaling patterns, with an increase in both spliced *XBP1*, CHOP transcription and induction of miR-494 (Supplementary Figure 1). Interestingly, TCN but not TG induced a biphasic expression of mature miR-494 with a slight increase at 1h and a more significant (∼30-fold) increase at 6 h. Moreover, primary miR-494 transcript expression mirrored that of the mature transcript in TG treatment but not TCN (Figure 1 and Supplementary Figure 1). More pathophysiological stimuli, hyperglycemia and oxidized LDL, also induced the primary miR-494 levels but less than the TCN and TG treatments (Supplementary Figure 3). It is unclear if these differences reflect differential regulation of miR processing pathways by these inducers. Indeed, the canonical miR processing protein Dicer has been shown to localize and interact with proteins in the ER (Pépin et al., 2012). Similarly, there are differences in primary and mature miR levels in response to our stressors. Interestingly, other studies have identified miRs and lncRNAs in this genomic region as being critical regulators of diabetic nephropathy (Kato et al., 2016). Specifically, Kato et al. showed that miRs in this cluster were induced during UPR in glomerular epithelial cells in a CHOP dependent manner. Our data suggests that in cultured ECs, ATF6 maintains the primary miR-494 levels and the all three transducers of ER stress ATF6/IRE1/PERK impact the mature miR-494 levels. Therefore, it is possible that different ER stress pathways can differentially impact miR-494 transcription, steady-state levels, stability as well as processing depending on the physiological or pathological contexts.

Our data from gain-and loss-of-function experiments show that miR-494 decreases ER stress in ECs (Figure 2). We observed that miR-494 induction in response to ER stress is acute and the kinetics of the DDIT3 and miR-494 are not directly reciprocal. miRs often downregulate targets over 24–48 h to regulate gene expression. Our experiments with the exogenous miRs function at better efficiency since the amount of miR available is higher than endogenous miR-494. The experiments where we inhibit endogenous miR-494 (e.g., Figures 2B,D,F) suggest that there is a role for the endogenous basal miR-494 in ER stress.

miR-494 is known to be oncogenic and drives tumor progression and drug resistance in specific cancer types including colorectal cancer (Zhang et al., 2018) and hepatocellular carcinoma (Pollutri et al., 2018; Zhang et al., 2019). Furthermore, miR-494 has been shown to attenuate ischemia reperfusion injury in the liver by regulating the PI3K/Akt signaling pathway (Su et al., 2017). We previously reported a unique role for miR-494 as a mediator of endothelial senescence by decreasing the MRN DNA repair protein complex. Other studies have shown that miR-494 is involved in vascular inflammation in atherosclerosis (van Ingen et al., 2019). Our data indicates a novel function of miR-494 in a complex pathological process.

Using a proteomics approach, we identified six putative miR-494 targets that are relevant during the ER stress response in ECs. Of these, survivin (*BIRC5*) has been previously shown to be a bona fide target of miR-494 in different disease models (Zhu et al., 2015; Yun et al., 2018). Interestingly, the cross-talk between ER stress and survivin has been shown to downregulate inflammatory genes in a mouse model of chronic ER stress in the colon (Gundamaraju et al., 2018). Consistent with our observations of a miR-mediated reduction in ER stress combined with a decrease in survivin (*BIRC5*) and markers of inflammation, Gundamaraju et al. (2018) demonstrated that pharmacological inhibition of survivin is comparable to ER stress inhibition and attenuates inflammatory gene expression. However, we found that genetic (siRNA) and pharmacological disruption of survivin (with YM155) did not alter the levels of DDIT3 in HUVECs. Therefore, decrease of survivin may not be sufficient to regulate ER stress at least as measured by DDIT3 levels. We propose this is likely due to a combinatorial effect due to multiple targets of miR-494 being involved in the ER stress response or an indirect effect on DDIT3 by unknown mechanism(s).

In summary, our work shows that miR-494 is induced during acute ER stress and ectopic overexpression functions to attenuate ER stress *in vitro*. Our observations elucidate a new potential mechanistic role for miR-494 in the ER stress response pathway in ECs and likely in other cell types. These studies offer opportunities to inspire new hypotheses to understand the link between miR activity and the response to stressors which influence cell fate decisions in many human diseases.

## Data Availability Statement

The datasets presented in this study can be found in online repositories. The names of the repository/repositories and accession number(s) can be found below: http://www.proteomexchange.org/, PXD019992.

## Author Contributions

NC, EF-B, CE-D, and SA designed the experiments. NC, EF-B, AB, and CE-D performed the experiments and analyzed the data. NC and SA wrote the manuscript. All authors reviewed and edited the manuscript.

## Conflict of Interest

The authors declare that the research was conducted in the absence of any commercial or financial relationships that could be construed as a potential conflict of interest.
