# Supplementary material for: MicroRNA-494 Regulates Endoplasmic Reticulum Stress in Endothelial Cells
Source: Front Cell Dev Biol. 2021 Jul 12;9:671461. doi: 10.3389/fcell.2021.671461 (PMC8311360; doi:10.3389/fcell.2021.671461)
Supplement: Supplementary file 1 [file Data_Sheet_1.PDF]

Chatterjee et al Supplementary Figure 1

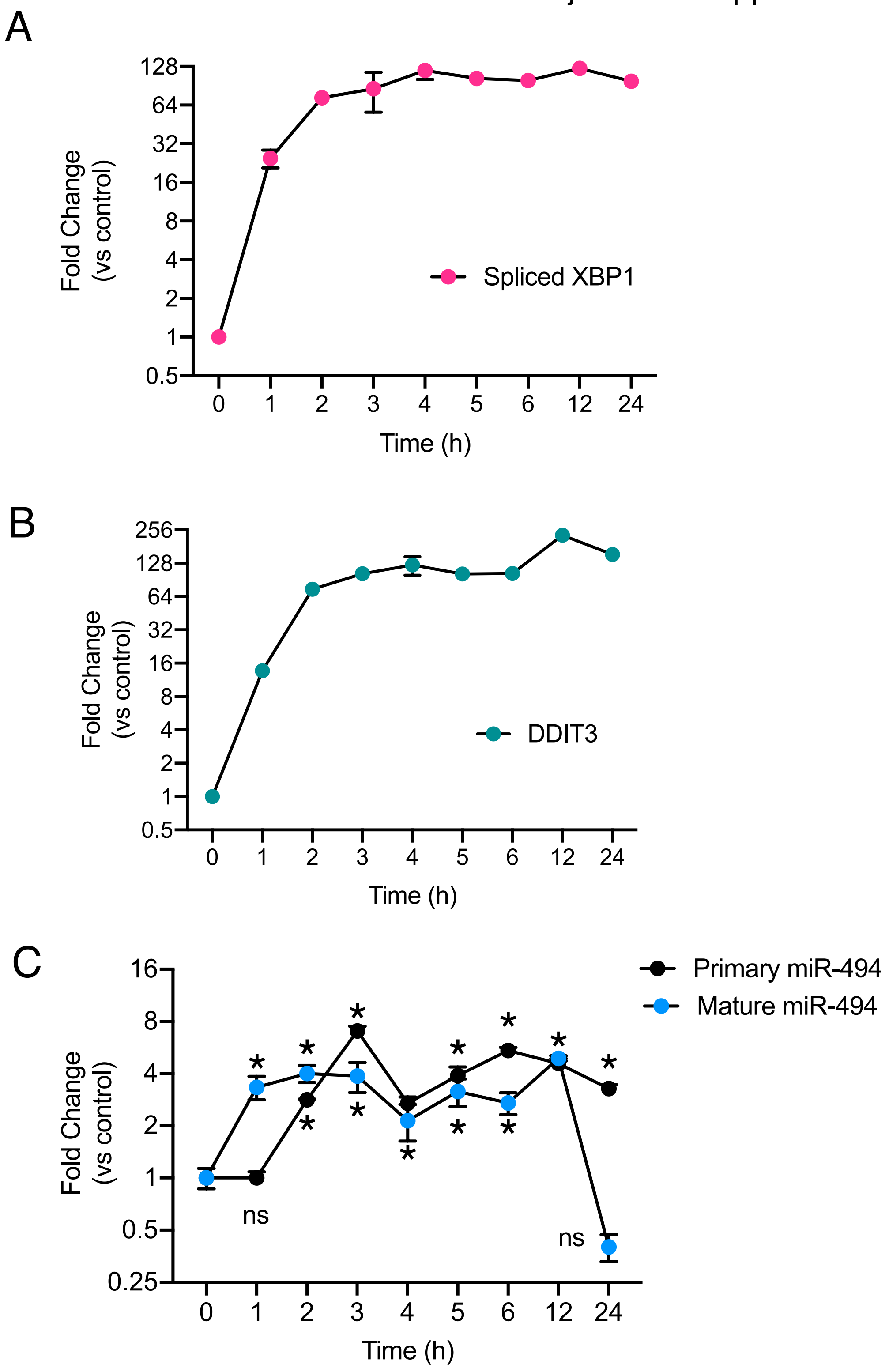

Supplementary Figure 1: Thapsigargin induced ER stress drives miR-494 expression. Relative mRNA expression of A) *sXBP1* and B) *DDIT3* (CHOP) mRNAs and C) primary and mature miR-494 in HUVECs treated with 0.1μM Thapsigargin. Gene expression is normalized to GAPDH or U6 and mean fold change compared to vehicle control or time 0h is shown. Graphs are representative of 1 of 3 biological replicates. Values indicate mean ± standard deviation. * P<0.05 using two-way ANOVA with Fisher's LSD test.

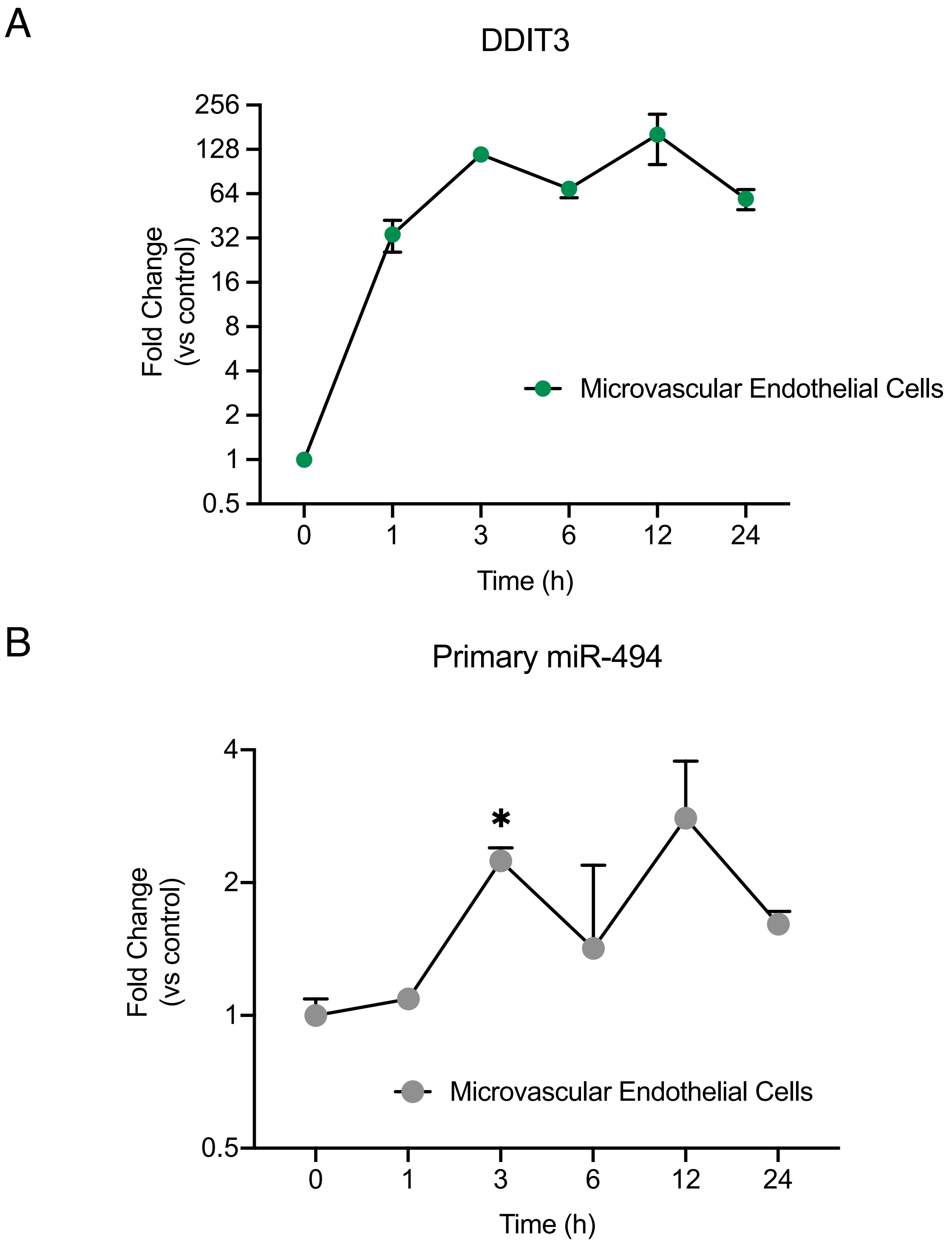

Supplementary Figure 2: ER stress induces expression of miR-494 in human microvascular ECs. A. Relative mRNA expression of *DDIT3* (CHOP) in HMVECs treated with 5µg/mL Tunicamycin. B) Relative expression of primary miR-494 treated with Tunicamycin. Gene expression is normalized to GAPDH and mean fold change compared to vehicle control or time 0h is shown. Graphs are representative of 1 biological replicate from 3 independent replicates where values indicate mean ± standard deviation. * $P<0.05$ using two-way ANOVA with Fisher's LSD test.

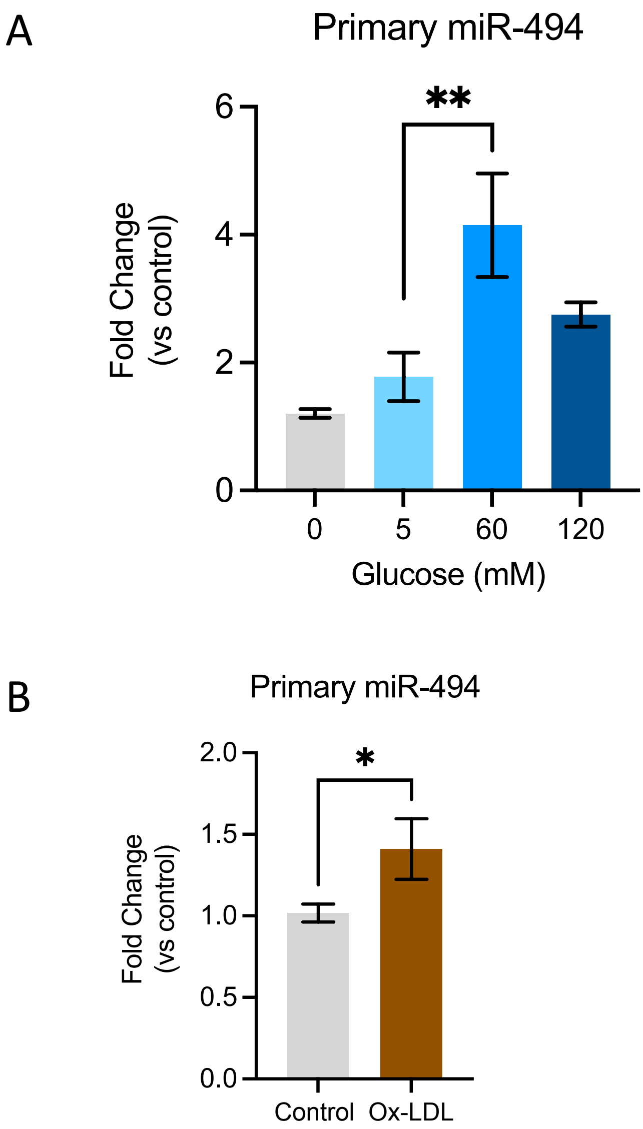

Supplementary Figure 3: miR-494 is induced by other modulators of ER stress.
HUVECs or were subjected to varying glucose concentrations (A) or treatment with Ox-LDL (100µg/ml) (B) 6h later primary miR-494 levels were assayed using qRT-PCR. Graph depicts mean fold changes of 3 biological replicates normalized to U6 small RNA levels.

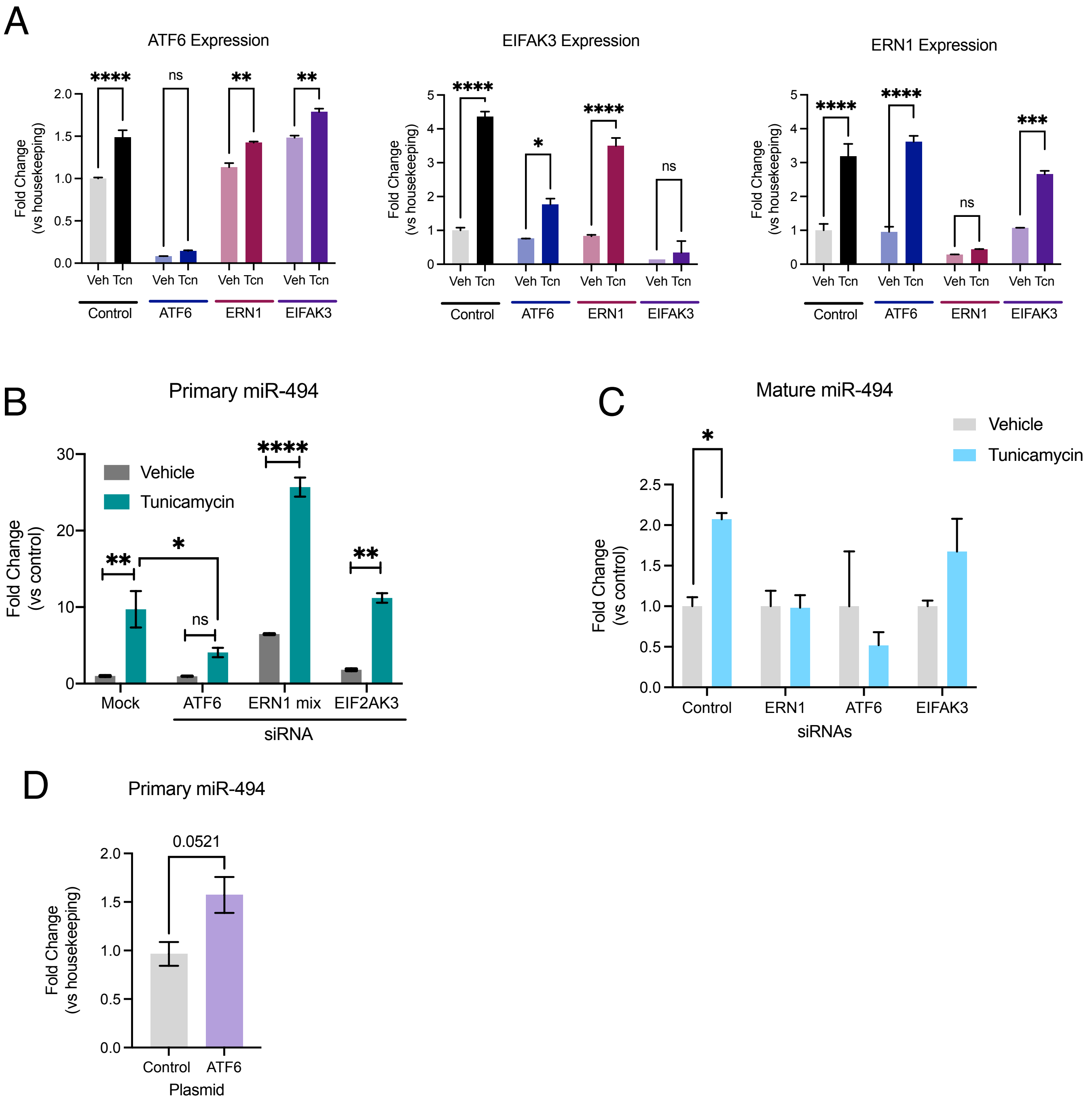

Supplementary Figure 4: Relative contributions of ATF6, ERN1 and EIFAK3 in miR-494 expression in response to ER stress.

HUVECs were transfected with indicated siRNAs. After 48h, cells were treated with vehicle or Tunicamycin (10µg/mL). Relative expression of A) ATF6, EIFAK3, ERN1 B) primary miR-494 C) mature miR-494 normalized to U6 is shown. D) HUVECs were transfected with a control vector or ATF6 plasmid. 24h later primary miR-494 levels were assayed using qRT-PCR. Mean fold change compared to vehicle control is shown. Graphs are representative of 1 biological replicate from 2 independent replicates where values indicate mean ± standard deviation. * *p*<0.05, ** *p*<0.01, **** *p*<0.0001 using two way ANOVA with a post-hoc Tukey's correction or two-tailed Student's T-test.

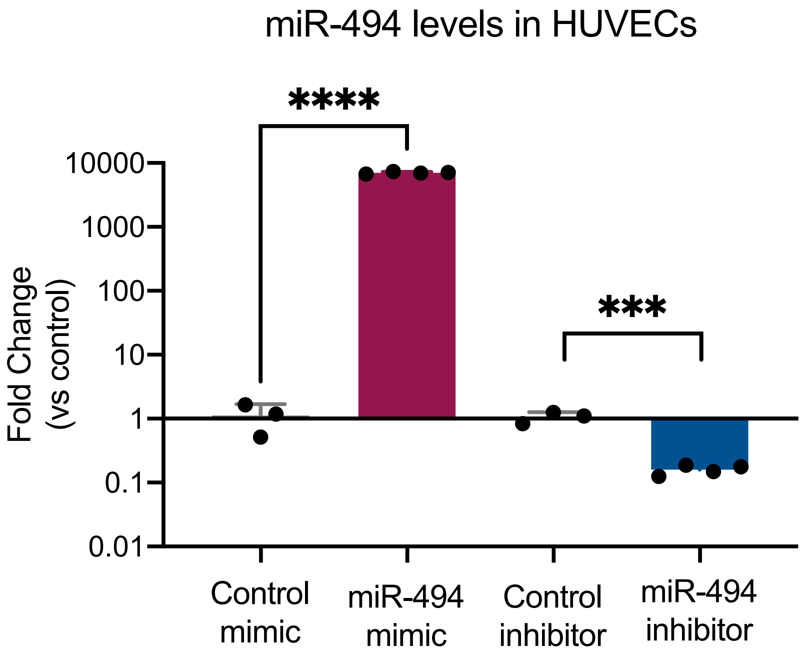

Supplementary Figure 5: Efficiency of miR-494 mimic and inhibitors in HUVECs. Cells were transfected with the indicated mimics or inhibitors. 24h later mature miR-494 levels were assayed using qRT-PCR. Graph depicts mean fold changes of 4 biological replicates normalized to U6 small RNA levels. Values indicate mean ± standard error. $P<0.001$ using two-tailed unpaired Student's T-test.

Commonly downregulated proteins in miR-494 & ER stress					
		miR-494 vs Control		Tunicamycin vs DMSO	
UniProt Gene Name	Other Gene Synonyms	FC	Adj P	FC	Adj P
G3BP1	G3BP	-1.60	2.43E-05	-1.43	4.53E-03
DUT	na	-1.54	2.84E-04	-2.08	2.04E-09
JUP	CTNNG; DP3	-1.54	4.45E-04	-1.87	1.48E-06
CDK1	CDC2; CDC28A; CDKN1;	-1.62	1.35E-04	-1.86	4.61E-06
HN1	ARM2	-1.60	7.32E-04	-1.70	4.09E-04
IL6ST	na	-1.59	1.69E-05	-1.72	3.81E-06
FSBP	na	-1.52	9.18E-03	-1.66	2.33E-03
TYMS	TS	-1.52	8.52E-04	-4.08	7.14E-29
CMSS1	C3orf26	-1.51	1.18E-04	-1.37	9.60E-03
SPC24	SPBC24	-1.58	5.57E-05	-2.46	1.18E-14
PRIM1	na	-1.77	2.43E-05	-2.16	1.26E-07
MINA	na	-1.89	1.18E-04	-2.31	1.81E-06
DHFR	na	-2.15	5.15E-04	-2.29	5.71E-04
GINS4	SLD5	-1.61	8.52E-04	-2.64	1.78E-11
UHRF1	ICBP90; NP95; RNF106	-1.80	3.07E-03	-10.48	8.88E-31
GMNN	na	-1.50	6.17E-03	-11.87	2.88E-62
CKS1B	CKS1	-2.08	3.81E-04	-2.39	8.42E-05
NME6	na	-1.85	7.32E-04	-2.45	3.20E-06
BIRC5	API4; IAP4	-2.18	1.75E-03	-22.34	3.02E-30
DNAH12	na	-1.78	4.94E-04	-1.90	3.61E-04
FZD6	na	-1.94	1.71E-04	-2.59	3.06E-07
BUB1B	BUBR1; MAD3L; SSK1	-1.92	7.94E-03	-4.18	4.45E-09
KIFC1	HSET; KNSL2	-3.46	4.74E-06	-12.33	1.33E-16

Supplementary Figure 6: List of common downregulated proteins from Fig 3A. Fold changes are mean of biological replicates compared to control treatments. Adj P values are from Benjamini-Hochberg correction. Only proteins with at least 1.5 fold decrease in miR-494 group were considered significantly downregulated.

Chatterjee et al Supplementary Figure 7

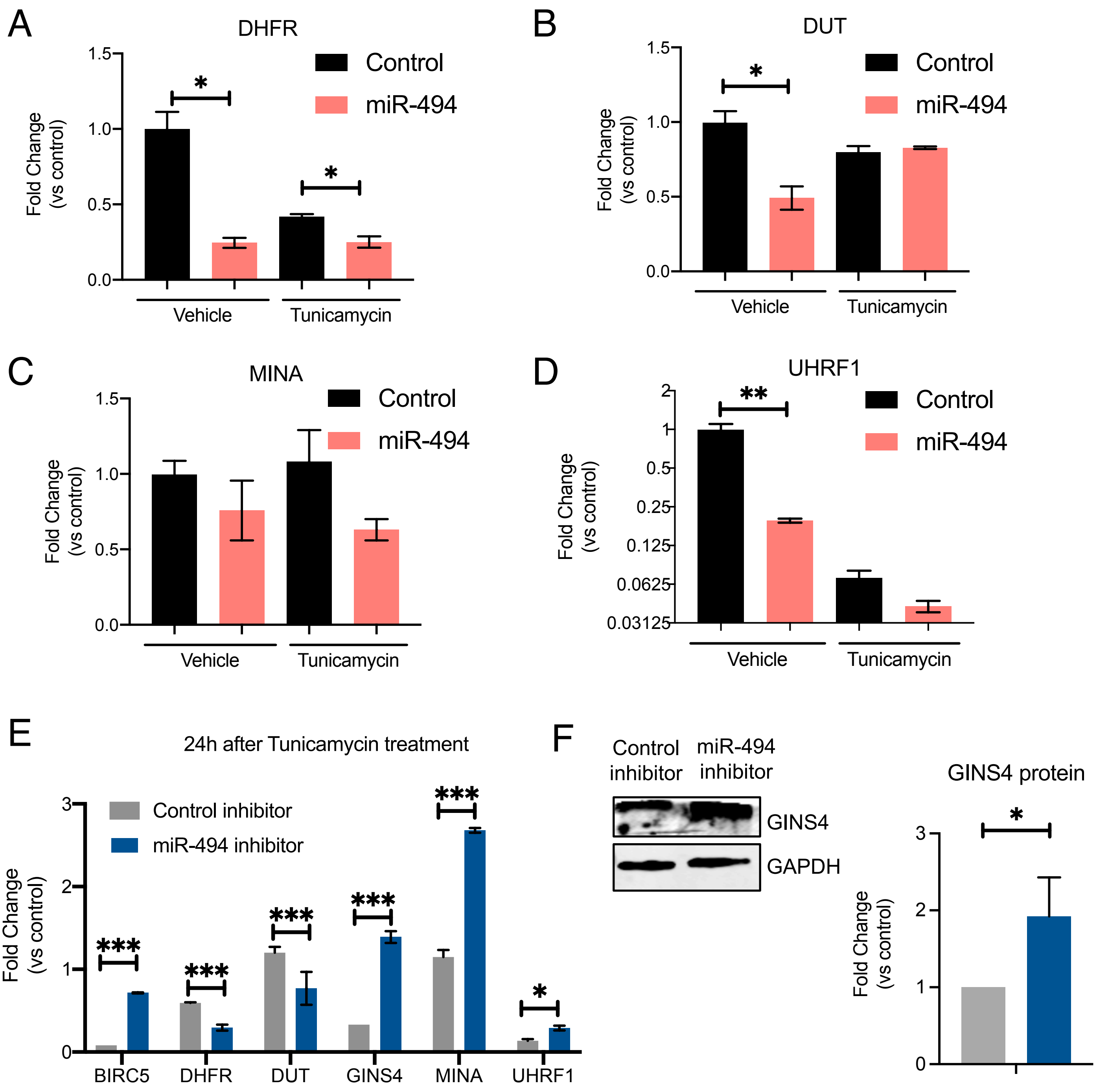

Supplementary Figure 7: miR-494 regulates several target genes in cell survival and DNA replication.
A-D) Fold-change (compared to respective controls) of mRNA levels as assessed by Quantitative PCR for the 4 targets that are downregulated in both Tunicamycin and miR-494 groups in Fig 3B. HUVECs were transfected and treated as described in 2A. E) HUVECs were transfected with either a control inhibitor or miR-494 inhibitor as shown in 2B. mRNA levels of indicated target genes were evaluated using qRT-PCR. Bars show mean + S.D. of one representative experiment out of three independent biological replicates. F) Western blot showing GINS4 protein levels 24h after transfection of a control inhibitor or miR-494 inhibitor. Bars show normalized band intensities (mean + S.D.) of three independent replicates. * P<0.05, ** P< 0.01, ***P<0.001 by two-tailed Student's T-tests comparing respective control groups.

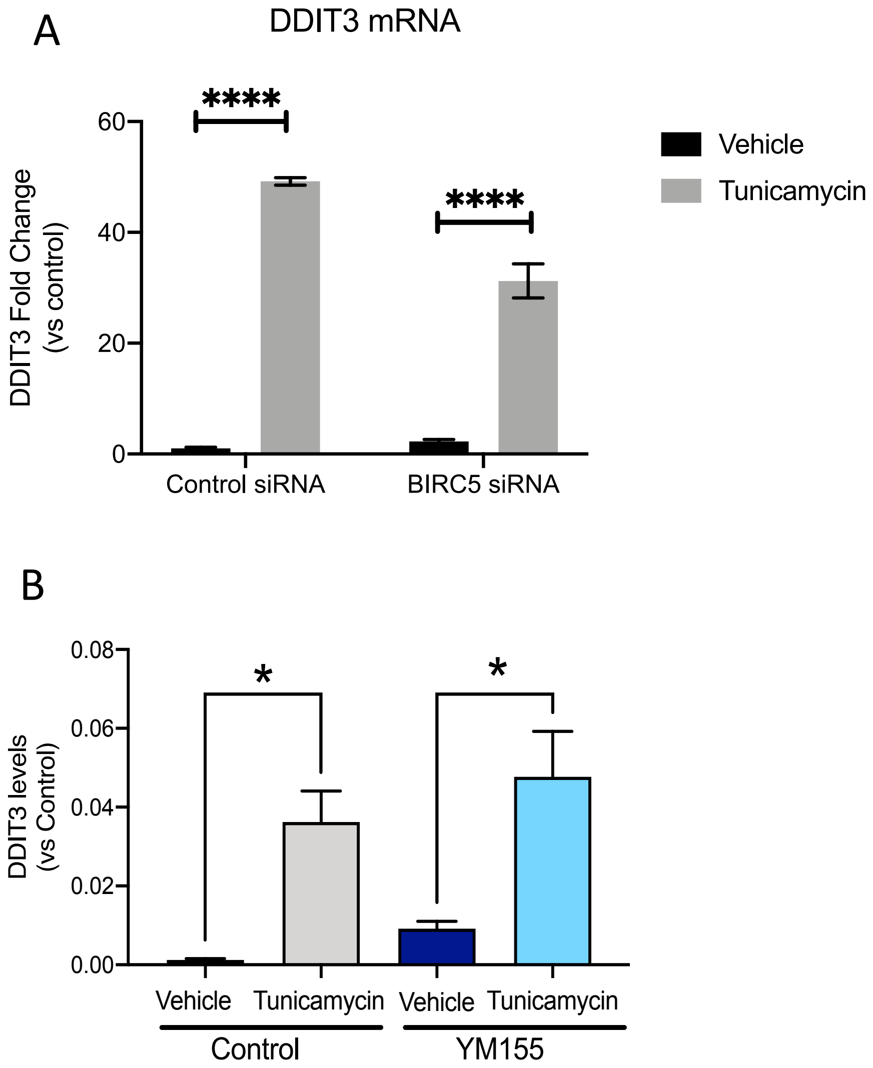

Supplementary Figure 8: Inhibition of Survivin does not diminish ER stress induced DDIT3. (A) HUVECs were transfected with siRNA targeting BIRC5. 24h later DDIT3 levels were assayed using qRT-PCR. Graph depicts mean fold changes (vs control treatment) of 3 biological replicates normalized to GAPDH RNA levels. B) HUVECs were treated with YM155, a small molecule antagonist of Survivin (50nM). 1h later ER stress was induced with Tunicamycin. RNA was harvested 24h later and DDIT3 levels were assayed using qRT-PCR. Graph depicts ΔΔ C_t values vs GAPDH.
